# Protection against Retrovirus Pathogenesis by SR Protein Inhibitors

**DOI:** 10.1371/journal.pone.0004533

**Published:** 2009-02-19

**Authors:** Anne Keriel, Florence Mahuteau-Betzer, Chantal Jacquet, Marc Plays, David Grierson, Marc Sitbon, Jamal Tazi

**Affiliations:** 1 Université Montpellier 2 Université Montpellier 1 CNRS, Institut de Génétique Moléculaire de Montpellier (IGMM), UMR5535, IFR122, Montpellier, France; 2 Laboratoire de Pharmaco-chimie, CNRS-Institut Curie, UMR 176 Bat 110 Centre Universitaire, Orsay, France; 3 Faculty of Pharmaceutical Sciences, University of British Columbia, Vancouver, British Columbia, Canada; Yale University, United States of America

## Abstract

Indole derivatives compounds (IDC) are a new class of splicing inhibitors that have a selective action on exonic splicing enhancers (ESE)-dependent activity of individual serine-arginine-rich (SR) proteins. Some of these molecules have been shown to compromise assembly of HIV infectious particles in cell cultures by interfering with the activity of the SR protein SF2/ASF and by subsequently suppressing production of splicing-dependent retroviral accessory proteins. For all replication-competent retroviruses, a limiting requirement for infection and pathogenesis is the expression of the envelope glycoprotein which strictly depends on the host splicing machinery. Here, we have evaluated the efficiency of IDC on an animal model of retroviral pathogenesis using a fully replication-competent retrovirus. In this model, all newborn mice infected with a fully replicative murine leukemia virus (MLV) develop erythroleukemia within 6 to 8 weeks of age. We tested several IDC for their ability to interfere ex vivo with MLV splicing and virus spreading as well as for their protective effect in vivo. We show here that two of these IDC, IDC13 and IDC78, selectively altered splicing-dependent production of the retroviral envelope gene, thus inhibiting early viral replication in vivo, sufficiently to protect mice from MLV-induced pathogenesis. The apparent specificity and clinical safety observed here for both IDC13 and IDC78 strongly support further assessment of inhibitors of SR protein splicing factors as a new class of antiretroviral therapeutic agents.

## Introduction

Retrovirus pathogenesis combines a whole array of mechanisms that can involve lytic, oncogenic, inflammatory or mutagenic processes that translate into a variety of diseases, including neoplasia, leukemias, immunodeficiencies, autoimmune syndromes, anemia, and thrombocytopenia and other hematopoietic disorders, neurodegenerative diseases and encephalitis, arthritis and osteopetrosis, etc. Murine leukemia virus (MLV) have been extensively used as models of retroviral pathogenesis because of the various pathogenic effects that can be selectively produced in mice. This diverse MLV-induced pathogenic outcome is dependent on a variety of parameters, including the virus and mouse strains or the age of infection [1]–[3]. When injected into mice of susceptible strains before 3 days of age, fully virulent strains of the replication-competent Friend MLV (F-MLV) invariably induce an erythroleukemia (EL) that results in the death of 100% animals, generally within 2 months after inoculation [4], [5].

The earliest phase of the disease has been shown to be directly dependent on the viral envelope glycoprotein (Env) [4], [5], while the latest phase involves more specifically retrovirus-mediated insertional mutagenesis governed by transcriptional promoting and enhancing properties of the U3 sequence in the MLV LTR [5]–[7]. In all retroviruses, Env is encoded by the main spliced retroviral mRNA. Other *cis*-acting sequences of the MLV genome, such as alternative or cryptic splice sites, have also been shown to play a specific role in the F-MLV leukemogenic process [3], [8]–[10]. Therefore, retroviral RNA metabolism, including transcriptional and splicing stages, is of paramount importance in the development of retroviral pathogenesis, in general, and F-MLV pathogenesis, in particular.

For all replication-competent retroviruses, replication and spreading depend on the production of two major RNA species, a full length mRNA and a single-spliced mRNA. The full-length mRNA can either be translated into the capsid Gag and the enzymatic Pol polyprotein precursors or be packaged into virions as a dimer to constitute the retroviral genome. The single-spliced mRNA encodes Env, the virus envelope glycoprotein which interacts with cellular receptors and which is essential for productive viral entry. Env expression is tightly dependent on the host splicing machinery and is a limiting requirement for virus spreading and pathogenesis. Mutations that affect MLV canonical or alternative splice sites have been shown to contribute to inefficient replication and altered pathogenic effects [3], [9], [10]. Therefore, inhibiting this single-splicing event offers a specific way to prevent retroviral spreading and pathogenesis.

Recently, we have reported the identification of several indole derivatives compounds (IDC) that mediate direct and selective interactions with members of the serine-arginine rich (SR) protein family of splicing factors [11], [12]. Certain IDC have been proven to be potent inhibitors of HIV-1 replication in cell culture through a selective action on exonic splicing enhancers (ESE)-dependent activity of individual SR proteins [12]. One such molecule, IDC16 has been shown to interfere with the SF2/ASF SR protein and production of HIV regulatory proteins and to compromise assembly of infectious particles [13]. However, no evaluation of IDC on retrovirus-mediated pathogenesis has yet been documented. Here, we have taken advantage of the F-MLV induced pathogenesis model in newborn mice to evaluate the efficiency of this new class of molecules at different stages of retrovirus infection and disease. We show now that different IDC differentially inhibit HIV-1 and MLV, most likely reflecting distinct requirement for cellular splicing factors. Thus, we found that IDC13 and IDC78, but not IDC16, prevented F-MLV replication both ex vivo and in vivo, by selectively altering single-splicing of the retroviral genome. Furthermore, we describe two IDC that also proved to be very efficient at protecting mice from MLV-induced pathogenesis by inhibiting early viral replication.

## Results

### IDC that can inhibit *ex vivo* replication of MLV

**Figure 1:**
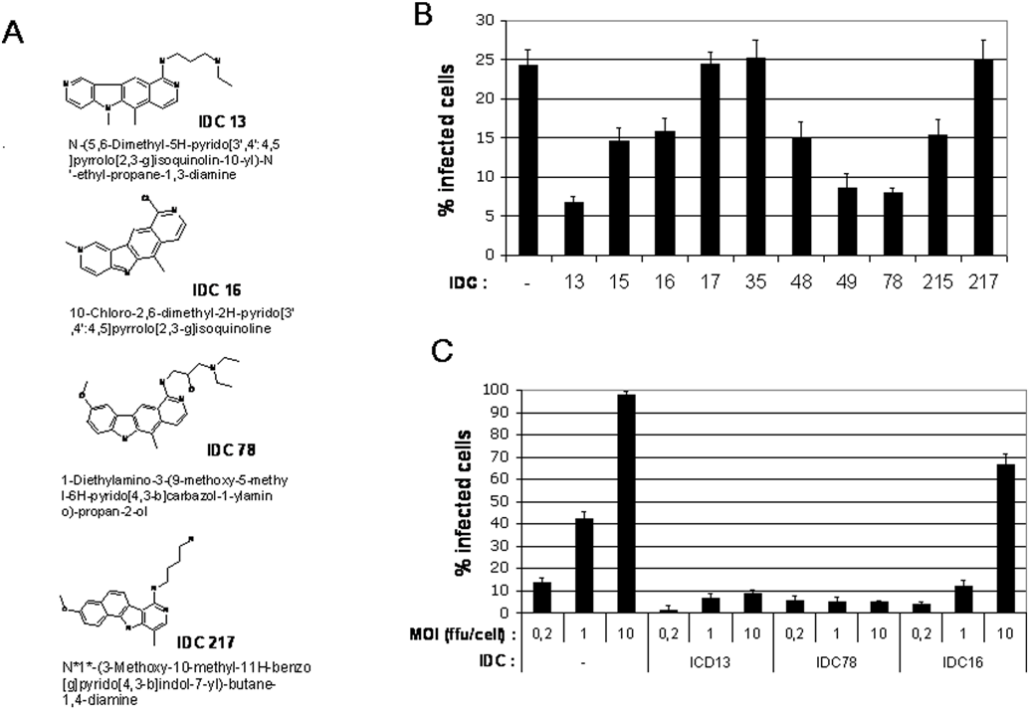
IDC can inhibit *ex vivo* replication of F-MLV in an MOI-dependent manner. A) Structure and formula of selected IDC compounds. B) Dunni cells were infected with Friend-MLV (strain 57) at a multiplicity of infection (MOI) of 0.5 foci forming unit (ffu)/cell in the presence of 1 µM of various IDC. Cells were stained 48 h post-infection with the H48 anti-F-MLV Env monoclonal antibody and analyzed by flow cytometry. C) Dunni cells were infected with increasing MOI of F-MLV (0.2, 1 or 10 ffu/cell) in the presence of 1 µM of IDC13, IDC78 or IDC16. Cells were stained 48 h post-infection with an anti-F-MLV Env antibody and analyzed by flow cytometry. The % of infected cells (i.e. cells stained by anti-Env) is indicated.

We first screened for IDC that could have an effect on *ex vivo* replication of MLV. Target murine cells were infected with a prototypic virulent strain of F-MLV at the low multiplicity of infection (MOI) of 0.5 focus-forming unit (FFU) per cell in the presence of various IDC. The number of infected cells was evaluated 48 h post-infection by flow cytometry, after staining with the H48 anti-F-MLV Env monoclonal antibody [14]. Among several IDC tested, IDC13 and IDC78 demonstrated the strongest inhibitory activity (Fig. 1A and Table S1). Interestingly, IDC16, which has been shown to inhibit efficiently *ex vivo* replication of HIV-1 [13], had a more moderate effect on F-MLV replication, suggesting that requirements for SR proteins vary with the retrovirus type.

We further evaluated the efficiency of this inhibition by testing increasing virus MOI (0.2, 1 and 10 FFU/cell) in the presence of IDC13, IDC78 or IDC16. In the absence of IDC, increasing MOI resulted in a non-linear increase in the percentage of infected cells (close to 100% of cells were infected at a MOI of 10 FFU/cell) (Fig. 1B). Treatment with IDC13 or IDC78 resulted in a strong decrease of F-MLV infection at all MOI tested, with up to 95% inhibition with IDC78 even at the highest MOI. In contrast, IDC16 did not prevent massive spreading of the virus when applied with the highest MOI, with a 35% inhibition of virus infection at the MOI of 10 FFU/cell (Fig. 1B).

The selective and highly most efficient inhibition of MLV infection observed here in cell culture with IDC13 and IDC78, but not IDC16, confirmed the distinctive requirements for cellular splicing factors by different types of retroviruses.

### IDC13 and IDC78 inhibit splicing of the MLV genome

**Figure 2:**
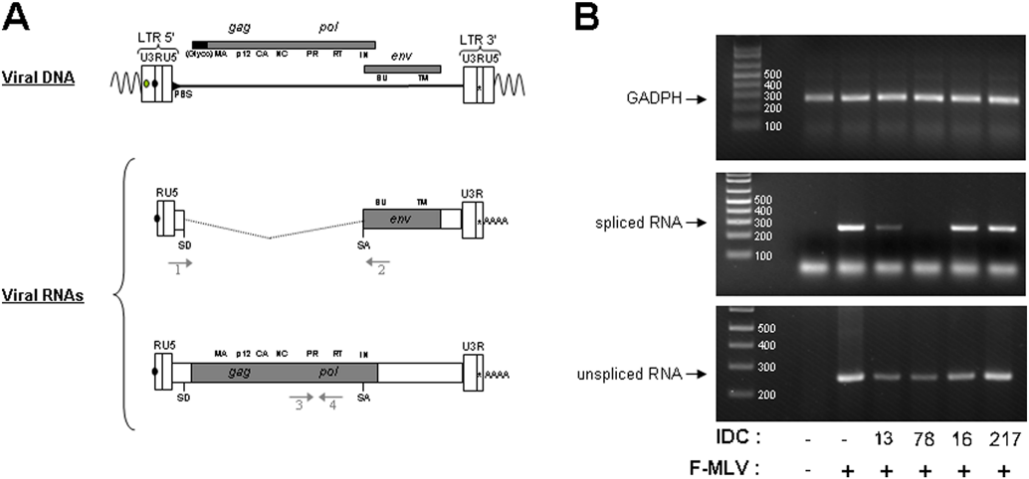
IDC13 and IDC78 alter splicing of F-MLV RNA. A) Schematic structures of integrated proviral DNA and spliced and unspliced F-MLV RNAs, including the position of the donor (SD) and acceptor (SA) splice sites. Arrows refers to the approximate positions of primers used to selectively amplify the spliced and unspliced viral product by PCR. Also noted are the *gag*, *pol* and *env* genes products. B) Dunni cells were infected with F-MLV at an MOI of 10 ffu/cell in the presence of 1 µM of IDC13, IDC78, IDC16 or IDC217. Total RNA was extracted 48 h post-infection, samples were treated with DNAse to remove any contaminating genomic DNA and used as a template for RT-PCR. Oligonucleotide primers specific for GADPH mRNA were used as internal control. The lower band migrating faster than spliced RNA (middle panel) corresponds to unhybridized oligos 1+2. The size markers in bp are indicated on the left of each panel.

In order to better understand the molecular mechanisms underlying the specific inhibition of MLV replication by some of the IDC, we analyzed the viral RNA content of infected cells. Dunni cells were infected with F-MLV in the presence of different IDC and total RNA was extracted and used as template for RT-PCR. We used two different sets of oligonucleotide primers that allowed us to discriminate between spliced and unspliced viral RNAs (Fig. 2A). As an internal control, RT-PCR was performed on mRNA from the *gadph* house-keeping gene.

Compared to untreated cells, accumulation of the PCR product corresponding to the spliced F-MLV RNA dramatically decreased upon treatment with IDC13 and IDC78 (Fig. 2B), while accumulation of the *gapdh* product did not decrease. Neither IDC16, mentioned above, nor IDC217, a compound that had no effect on all splicing substrates tested [12], had detectable impact on F-MLV splicing (Fig. 2B). Altogether, these results indicated that inhibition of F-MLV replication by certain IDC appeared directly associated with their ability to specifically inhibit viral RNA splicing, an event required for expression of the viral Env glycoprotein. However, we observed that the significant decrease of spliced product observed after IDC13 and IDC78 treatment was not compensated by a corresponding increase of unspliced F-MLV RNA. Instead, we noted that IDC13, IDC78 and also IDC16 affected, albeit to a lesser extent for the latter, accumulation of unspliced viral RNA (Fig. 2B, lower panel).

These results suggested that IDC13 and IDC78 inhibited F-MLV replication by altering viral RNA splicing, but that other pathway(s) governing RNA accumulation, such as transcriptional levels, RNA trafficking and/or RNA stability, could also be altered.

### IDC13 and IDC78 protect mice against F-MLV pathogenesis

We then examined the impact of IDC treatment on the *in vivo* replication and pathogenesis of F-MLV after inoculation of newborn mice. When injected in susceptible mice strains before the age of 3 days, this virus very reproducibly induces erythroleukemia (EL), resulting in the death of 100% animals within 2 months [4], [15].

**Figure 3:**
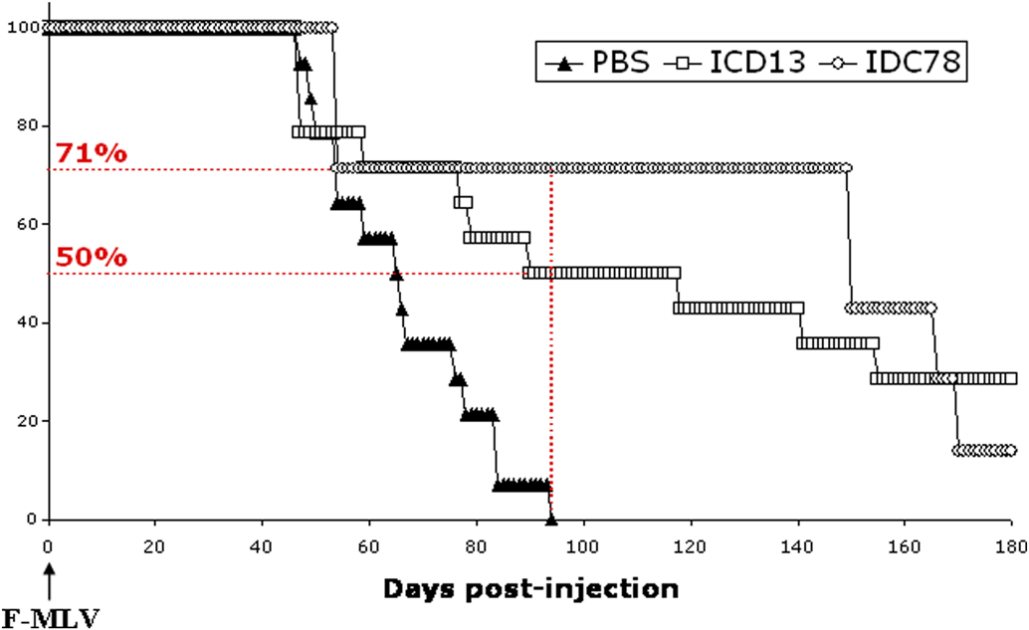
IDC13 and IDC78 protect mice against MLV-induced erythroleukemia. Newborn Swiss mice were injected intra-peritoneally with F-MLV together with either PBS, IDC13 or IDC78. Development of F-MLV induced erythroleukemia was monitored by occurrence of severe anemia and splenomegaly. Evaluation of pathogenicity was plotted as % of survival in the different groups. The % of survival in the two groups of IDC treated mice, at the time when all mice of the untreated group were dead, are indicated.

In order to evaluate the effect of IDC on MLV-induced EL, newborn mice were injected with 1 ffu of F-MLV (strain 57) and treated with IDC13, IDC78 or PBS, used as inoculation control. The disease parameters that were followed were anemia, splenomegaly or other organ enlargement, general aspect and survival. Even though previous reports on F-MLV pathogenesis used >10^4^ FFU/mouse, we show here that a viral input as low as 1 ffu was sufficient to induce EL in newborn mice. Indeed, 100% of control mice (treated with PBS) developed splenomegaly and died from EL within 95 days after inoculation of this dose of F-MLV (Fig. 3). Severe anemia could also be detected in these animals (data not shown). In contrast, mice treated with compound IDC13 (n = 14) or IDC78 (n = 7) showed a significantly increase in the latency of MLV-induced EL. At the time when all control mice were dead, IDC-treated mice showed a 50% or 71% rate of survival, respectively (Fig. 3). Within these two groups, several mice even survived, without any detectable symptom, up to 506 days before being sacrificed.

### IDC13 and IDC78 inhibit *in vivo* replication of F-MLV

To determine whether the low virulence observed in mice treated with IDC13 and IDC78 was due to a block of F-MLV *in vivo* replication, plasma from treated and non-treated mice were assayed for early infectious virus content (13 days post-inoculation). The quantification of plasmatic viremia was carried out after infection on highly susceptible Dunni cells using a focal immunostaining assay (FIA) [16].

**Figure 4:**
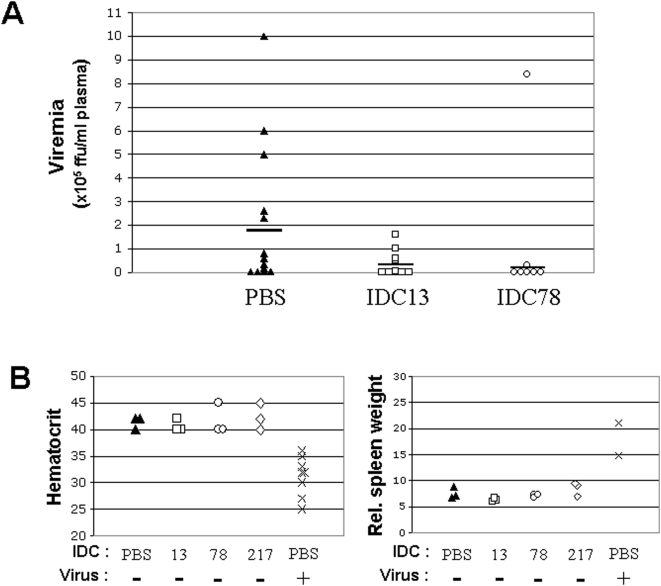
IDC13 and IDC78 do not significantly affect normal erythropoiesis while inhibiting *in vivo* F-MLV replication. A) Early plasmatic viremia was measured in mice infected with F-MLV and treated with each indicated IDC or with PBS. Values indicate the plasmatic virus titer of individual mice, as measured by infection of Dunni cells by serum dilutions recovered at 16 days of age. Mean values are indicated as a bar for each group. B) Newborn Swiss mice were injected only with IDC (or PBS as a control) and evaluated between 16 and 21 days of age for indicators of erythropoiesis, i.e. hematocrit as previously described [5], [15] (left panel) and relative spleen weight calculated as (spleen weight/mouse weight)×1000 (right panel). For comparison, the values measured for F-MLV injected mice, showing signs of F-MLV induced early anemia, are included in the graphs.

In control mice, plasmatic viremia 13 days after inoculation ranged from undetectable to 10^6^ ffu/ml, with a mean value of 1.9×10^5^ ffu/ml (Fig. 4A). Viremia of IDC13-treated mice ranged between undetectable and 1.6×10^5^ ffu/ml with a mean value of 3.7×10^4^ ffu/ml. In IDC78-treated mice, viremia varied between undetectable and 3×10^4^ ffu/ml, with the exception of 1 mouse that had a viremia of 8.4×10^5^ ffu/ml. Furthermore, we observed that there was a correlation between a lower plasmatic viremia and increased latency of disease in IDC-treated mice (data not shown). Altogether, this indicated that lower virulence of F-MLV, observed in IDC-treated mice, was likely due to inhibition of virus replication during the early phase of the disease.

In order to further assess whether resistance to F-MLV-induced erythroleukemia in IDC-treated mice was indeed due to inhibition of early virus dissemination, and not to a toxic effect leading to reduction of target cells, we measured the direct effect of these compounds on erythroid differentiation *in vivo*. Newborn mice were injected with compounds IDC13, IDC78 or IDC217 (or PBS as a negative control) and followed both for hematocrits between 16 to 21 days of age and for spleen enlargement as an indication of compensatory splenic activity. There was no significant difference of either hematocrit or relative weight values between the 4 groups (Fig. 4B), indicating that all three IDC, regardless of their virus inhibitory activity, had no direct detectable effect on erythroid differentiation in mice.

To gain a better understanding of the level of transcript variation in mice treated with IDC78, we performed a differential analysis of total splenocyte RNA extracted from IDC-treated mice spleens as compared to PBS-treated mice. Probes were prepared from pooled RNA samples to hybridize to 〈〈 Affymetrix GeneChip® Mouse >Exon 1.0 ST Array 〉〉. Out of 6000 genes that were detected with high confidence only 52 showed a gene expression level fold change (treated vs untreated) comprised between 1.5 and 3 (Figure S1). A likely explanation for little changes in the expression of endogenous gene compared to F-MLV could be that the viral RNA has to escape the splicing machinery during later stages of infection to produce viral particles containing full length unspliced pre-mRNA, whereas most cellular genes have constitutive exons that contain redundant binding sites for SR proteins.

Altogether, our data suggest that some IDC, which are members of a new class of SR protein inhibitors, can protect animals from retroviral pathogenesis, partly due to alteration of splicing of the retroviral genome leading to inhibition of early viral replication.

## Discussion

Expression of retroviral proteins, thereby retroviral replication and spreading, rigorously depends on the splicing of the viral genome. Here, we identified new IDC that altered splicing of the retroviral genome and conferred protection from MLV-induced retroviral pathogenesis. Interestingly, we found that IDC action could be selective depending on the retrovirus used. Thus, we have previously shown that IDC16 is a powerful inhibitor of HIV-1 replication assayed in cell cultures [12], [13], while here we found it inefficient at inhibiting MLV replication. Conversely, IDC13 and IDC78, which have a strong protective effect against F-MLV pathogenesis, were found to affect neither splicing nor replication of HIV-1. Therefore, the IDC emerge as new antiviral agents that can selectively target splicing events essential for the viral life cycle.

While the exact mechanism responsible for the selective impact of IDC remains to be elucidated, the targeting of SR proteins by these compounds is likely to involve post-translational steps when modifications and/or interaction of SR proteins with specific and/or constitutive splicing factors take place. Indeed, IDC have been shown *ex vivo* to bind directly to the RS domain of SR proteins and thereby impede its phosphorylation. Several IDC have been shown to prevent phosphorylation of RS domains by topoisomerase I and to a lower extent by Clk/Sty kinase, a modification known to be required for ESE-dependent splicing [17]. It is therefore possible that the effect of the drug involves modulation of the phosphorylation status of specific SR proteins. Another potential level of action of IDC is viral RNA trafficking. In favor of this is the fact that several SR proteins are able to shuttle between the nucleus and the cytoplasm [18], [19]. This property, which appears to be linked to the ability of SR proteins to interact with the nuclear import protein transportin-SR [20], [21], is also regulated by phosphorylation [22]. Since phosphorylation affects both splicing activity and sub-cellular trafficking of SR proteins [17], it would be interesting to evaluate the effect of IDC treatment on SR protein phosphorylation and retroviral RNA trafficking by SR kinases [11], [12], [23]. Treatment of cells with IDC may modulate both processes and act synergistically to modify MLV RNA splicing and/or export. This dual impact may explain the reduced accumulation of full-length MLV RNA also distinctively observed with IDC13 and 78.

Drugs interfering with the phosphorylation level of SR proteins and/or interaction with cellular factors are expected to modify the alternative splicing pattern of several genes. Such drugs which target most, if not all SR proteins, likely exhibit a significant cytotoxicity and are therefore less compatible with long term treatments. Conversely, compounds inactivating SR proteins with a higher selectivity should prove to be less toxic and more adapted to treat diseases in which the SR protein to be inactivated is well characterized. In this respect, it is encouraging that treatment of newborn mice with several IDC did not detectably alter the splicing profile of endogenous splenic genes, as revealed by a comprehensive exon microarray designed to detect alteration of splicing events (Figure S1). Also, the minimal side effects observed in our animal model further confirm that IDC, unlike deletion of the gene encoding SR proteins, are selective for factors or functions that can apparently be substituted by other SR protein family members. IDC13 and IDC78 but not IDC16 increased life expectancy of mouse we tested, whereas SR protein depletion is detrimental for survival [24]. Therefore, as used here, it is unlikely that IDC impede constitutive functions of SR proteins in gene expression, such as mRNA export [18], [22], [25], [26], mRNA stability [27], stimulation of mRNA translation [26] or maintenance of genomic stability [28], [29].

Despite the fact that IDC were initially selected by ex vivo experiments performed with very simple splicing substrates [12], these molecules reveal to inhibit splicing events in vivo with good specificity. Indeed, some of the IDC we tested have been shown to be potent inhibitors of HIV-1 production in cells chronically infected by the virus. Since HIV-1 alternative splicing events are known to be regulated by several members of the SR proteins family [30], inhibition of splicing by IDC is a likely mechanism for the remarkable antiviral activities exhibited by these molecules in cell culture systems. In agreement with this prediction, one selected molecule, IDC16, that has been shown to interfere with ESE activity of the SR protein splicing factor SF2/ASF, inhibits HIV1 replication of macrophage- and T cell–tropic laboratory strains, clinical isolates, and strains with high-level resistance to inhibitors of viral protease and reverse transcriptase [13]. The study presented here addresses for the first time the antiretroviral potential of such compounds in an in vivo model of retroviral replication and potent pathogenic effect and further confirms the effectiveness of IDC as antiviral agents. Interestingly, we found that the IDC that exerted the maximum effect on HIV, lentivirus genus, and MLV, gammaretrovirus genus, were distinct. It is still unclear whether these differences were due to the use of a different array of SR proteins involved in RNA metabolism by gammaretroviruses, which rely mainly on single-splicing events, or lentiviruses whose replication and spreading is tightly dependent on multi-splicing events that govern the formation of key regulatory proteins [31]. Alternatively, distinct cell tropism and tissue distribution for the two types of viruses may play a role in cell type-specific splicing events. In this view, the recent description of the cell-specific role of hnRNPL in alternative splicing of CD45 in activated T cells [32] brings new clues on potential mechanisms that underlie cell-specific effects of IDC. Here, we describe a genetically malleable in vivo model which can help further identification of SR protein that play a specific role in regulating retroviral splicing, replication and thereby infection, spreading and dissemination. Such studies coupled with the testing of IDC class of inhibitors should help the development of new therapeutic antiviral agents.

## Materials and Methods

### Cells

*Mus Dunni* tail fibroblasts (Dunni cells) were grown as monolayer cultures in Dulbecco modified Eagle's medium supplemented with 10% heat-inactivated fetal calf serum, 2 mM L-glutamine, 100 U/ml penicillin, 100 µg/ml streptomycin and 1% non-essential amino acids.

### Virus stock

The Friend-MLV prototype strain 57 has been reported earlier [15]. For preparation of viral stocks, supernatants were collected from chronically infected Dunni cells led to confluent monolayer. Titration of viral stocks was performed by focal immunostaining assay (FIA) [16]. Briefly, Dunni cells were infected with serial dilutions of viral stocks in the presence of polybrene (2 µg/ml). After 2 days of infection, cells were labeled with the H48 anti-F-MLV Env monoclonal antibody [14] and the titer was determined by the number of foci per well, which varies linearly with the viral input in this assay.

### *Ex vivo* replication assay

Dunni cells were seeded on 96-well plates (5×10^3^ cells/well) the day before infection. Cells were infected with F-MLV 57 at an MOI of 0.2, 1 or 10 ffu/cell in the presence of 1 µM of various indole derivative compounds and 2 µg/ml of polybrene. Cells were stained 48 h p.i. with the H48 anti-Env monoclonal antibody and an anti-mouse IgG serum coupled to FITC (Sigma). Cells were then detached in PBS-5 mM EDTA and analyzed by flow cytometry (FACSCalibur, Becton Dickinson).

### RNA analysis

Cells were infected with F-MLV 57 at a MOI of 10 ffu/cell and cultured in 6-well plates for 2 days at 37°C. Cells were washed twice with phosphate-buffered saline, and total RNA was extracted from cell pellets with TriReagent (Sigma) according to the manufacturer's instructions. The samples were treated with RNase-free DNase (RQ1, Promega) to remove DNA contamination. Cellular RNA concentrations were quantitated by measuring optical absorption at 260 nm.

RT was performed with the first strand cDNA synthesis kit (GE Healthcare). Five µg of cellular RNA sample were denatured at 65°C for 10 min and chilled for 5 min at 4°C before reverse transcription was performed for 1 h at 37°C in a 15 µl reaction volume containing 200 ng of oligo(dT)18, 10 mM deoxynucleoside triphosphate (dNTP), 10 mM dithiothreitol DTT, RNase/DNase-free BSA, RNAguard™ and recombinant MLV RT. One-tenth of each reaction mixture was used as the starting material for the different PCRs.

Two combinations of oligonucleotide pairs were used to detect specific F-MLV transcripts. The oligonucleotide sequences were as follow (with the sizes of the amplified products indicated in parentheses): oligo1 5′-CGTGGTCTCGCTGTTCCTTGG-3′ and oligo2 5′-GCGGACCCACACTGTGTC-3′ (259 bp) for unspliced F-MLV RNA detection and oligo3 5′-GATATCGGGCCTCGGCCAAG-3′ and oligo4 5′-AAACAGAGTCCCCGTTTTGG-3′ (250 bp) for spliced RNA. Primers to amplify GAPDH were as follows: (sense) 5′ -GCTCACTGGCATGGCCTTCCGTG-3′ and (antisense) 5′-TGGAAGAGTGGGAGTTGCTGTTGA-3′ (200 bp). PCRs were carried out with 0.2 mM dNTP mix, 3 mM MgCl2, PCR buffer, 1.5 U of Taq DNA polymerase (InVitrogen) and 200 ng of each of the sense and antisense primers. Denaturation, annealing, and extension were performed at 94, 58, and 72°C, respectively. PCRs were performed on a RoboCycler Gradient 96 thermocycler (Stratagene) with 29 cycles. These cycles were preceded by a 5-min denaturation at 94°C and terminated by a 10-min extension at 72°C. Amplified samples were electrophoresed on agarose gel, stained with ethidium bromide and bands were quantified by FluorImager.

### *In Vivo* experiments

Newborn OF-1/Swiss mice were injected intra-peritoneally before 3 days of age with 1 focus-forming unit (ffu) of F-MLV strain 57 together with either PBS or IDC13 or IDC78 (0,2 µg/g of weight, diluted in PBS). Mice were subsequently injected with either PBS or with IDC13 or IDC78 (0,2 µg/g of weight, diluted in PBS) 6 h after the first injection and every other day during 10 days.

Hematocrit and spleen enlargement, two indicators of erythroleukemia, were monitored weekly from day 16 of age. Splenomegaly was monitored by palpation and blood was collected by retro-orbital puncture on animals anesthetized with methoxyflurane vapor (isofluorane) to measure their hematocrit. All animal procedures were carried out according to the European Communities Council Directive (86/609/EEC) and Convention (ETS123) issued in 1986. Mice were routinely monitored for evidence of disease and moribund mice were sacrificed.

For evaluation of IDC effect on erythropoiesis, three-days old OF-1/Swiss mice were injected intra-peritoneally with either PBS or IDC78 (0,2 µg/g of weight, diluted in PBS) and sacrificed at 4 days old. Spleens were dissected out and weighed. Total RNA from frozen spleens was extracted using the Trizol (according to the manufacturer instructions) and taken up in 10 mM Tris-HCl pH 8, 0.1 mM EDTA.

### Measurements of viremia in mice

Mice serum was obtained from blood collected on 16 days old mice by centrifugation at 400 g for 5 min. Viremia was measured by FIA as described earlier [16]. Briefly, 2×10^4^ Dunni cells were plated on 12-well plates and infected the day after with serial dilutions of mice serum in the presence of 2 µg/ml of polybrene. Cells were fixed 2 days latter with a 4% solution of paraformaldehyde and stained with H48 and a FITC-coupled secondary antibody as mentioned above. The number of foci, representing the number of ffu contained in the volume of serum used to infect Dunni cells, was counted and viremia was calculated in ffu/ml.

## Supporting Information

Table S1 Supplemental data to Figure 1
(0.05 MB DOC)

Figure S1 We have used 〈〈 Affymetrix GeneChip® Mouse Exon 1.0 ST Array 〉〉. The annotated probes are fully described in http://www.affymetrix.com/. The hybridization procedure and data analysis are described in https://www.affymetrix.com/support/downloads/manuals/wt_sensetarget_label_manual.pdf and http://www.affymetrix.com/support/technical/whitepapers/exon_alt_transcript_analysis_whitepaper.pdf, respectively. Sketch normalization was performed using the Expression Console software from Affymetrix. Background was calculated and subtracted from main probe intensities using the antigenomic probes as described (Clark TA, Schweitzer AC, Chen TX, Staples MK, Lu G, et al. (2007) Discovery of tissue-specific exons using comprehensive human exon microarrays. Genome Biol. 8(4): R64.). Bad quality probes including probes with high DAPG p-value in both experimental conditions were not selected. Gene expression level mean ratio (treated vs. control) was calculated by summarizing individual probe ratio and normal law p-values were calculated with the mean ratio and the corresponding standard deviation. Only 8 genes were identified with a gene expression level fold change >2 and with a p-value<0.05. Only 45 were identified with a gene expression level fold change >1.5 with a p-value<0.05. They are shown in red in the table.
(2.75 MB XLS)
