# Supplementary material for: Protection against Retrovirus Pathogenesis by SR Protein Inhibitors
Source: PLoS One. 2009 Feb 19;4(2):e4533. doi: 10.1371/journal.pone.0004533 (PMC2640060; doi:10.1371/journal.pone.0004533)
Supplement: Table S1 — Supplemental data to Figure 1 (0.05 MB DOC) [file pone.0004533.s001.doc]

**Table S1**: Chemical structure and formula of IDC compounds that scored negative at inhibiting replication of F-MLV *ex vivo*

| N° | Structure | Formula |
| --- | --- | --- |
| **IDC15** |  | **C23H29N5** |
| **IDC17** |  | **C23H28N4O** |
| **IDC35** |  | **C21H24N4O** |
| **IDC48** |  | **C22H27N5** |
| **IDC49** |  | **C25H32N4O** |
| **IDC215** |  | **C24H31N5O** |
